# Use of Citrated Whole Blood for Point-of-Care Viscoelastic Coagulation Testing in Dogs

**DOI:** 10.3389/fvets.2022.827350

**Published:** 2022-03-07

**Authors:** Whitney York, M. Ryan Smith, Chin-Chi Liu

**Affiliations:** Department of Veterinary Clinical Sciences, School of Veterinary Medicine, Louisiana State University, Baton Rouge, LA, United States

**Keywords:** viscoelastic, coagulation testing, point of care, VCM Vet, resting time

## Abstract

**Background:**

A new, portable bedside coagulation monitor (VCM Vet) has provided a user-friendly, cartridge-based method to perform viscoelastic testing. However, the use of native whole blood limits the time to analyze the sample to minutes. The objective of this study is to assess whether citrated whole blood can be utilized with the cartridge-based system and whether the results are comparable to those of native whole blood. A secondary objective is to assess the viability of citrated whole blood results after up to 4 hours of resting.

**Methods:**

The study population consisted of 10 healthy mixed breed dogs. Whole blood samples were collected *via* jugular venipuncture. Blood was immediately transferred to the VCM test cartridge for native whole blood control group analysis per the manufacturer's instructions, and the remainder was used to fill two 3.2% sodium citrate vacutainer tubes. Test group analysis was performed on samples from each tube concurrently after a rest period of 30 min (baseline), 2 h, and 4 h. Citrated whole blood samples were recalcified for analysis immediately prior to introduction into the test cartridge. Data was recorded for all reported parameters. Results from the citrate groups were compared to the control group and to the citrated baseline to assess for differences. Overall results were compared using mixed ANOVA models. Where found, specific differences were evaluated using Tukey's test. Within-sample variation was investigated and reported as median (range). A *p* < 0.05 was considered significant.

**Results:**

Samples were obtained for a total of 10 control runs and 20 citrated whole blood runs. Comparison of controls to the citrated test groups revealed significant differences in CT (*p* < 0.001) and MCF (*p* < 0.002). There were no significant differences between test groups compared to citrated baselines for any parameter. Selected median coefficients of variation were 6.8% (0–68.8%) for CT, 2.4% (0–19.46%) for alpha angle, 3.2% (0–27.4%) for MCF, and 0% (0–16.3%) for 45-min LY45.

**Conclusion:**

Citrated whole blood samples can be used with the VCM Vet device; however, new reference intervals for use with citrated whole blood will be required. Results using citrated whole blood samples are not significantly different from baseline after up to 4 h of resting.

## Introduction

Viscoelastic testing provides a more comprehensive evaluation of coagulation compared to traditional factor-based tests (1, 2). Traditional methods of viscoelastic testing, including thromboelastography (TEG) and thromboelastometry (TEM), have historically been time intensive and subject to sample handling and lab errors. Traumatic venipuncture, sample collection site, sample resting time, resting temperature, patient age, and patient sex have all been shown to affect results (2–4). Consequently, the clinical utility of the information produced have borne some scrutiny due in large part to variability of results (5).

Recently, a new portable bedside viscoelastic coagulation monitor (VCM Vet™, Entegrion, Durham, NC) has been validated for use in veterinary species (6, 7). This device provides a user-friendly, cartridge-based method to perform viscoelastic testing, which can significantly reduce the points of error that are typically associated with viscoelastic testing. However, the use of native whole blood (NWB) limits the time to begin analysis of the sample to minutes (8). The clinical situation may not always be conducive to such time constraints and sample requirements, especially in cases where venipuncture is challenging and/or *post-hoc* analysis is desired. The objective of this study was to assess whether citrated whole blood (CWB) can be utilized with this cartridge-based system and whether the results are comparable to those of NWB. A secondary objective was to assess the viability of CWB results after up to 4 hours after blood collection. We hypothesized that results obtained from CWB samples would differ from NWB samples and that CWB results would be affected by increasing rest periods.

## Materials and Methods

### Animals

Ten clinically normal intact female mixed breed dogs were used for this study. The dogs were part of an institutional research colony with a mean age of 3.9 years (range 2.5–4.0) and mean weight of 11.8 kg (range 9.6–13.2). Dogs were deemed clinically normal based on physical examination and clinical history. The dogs were not subjected to any medications or treatments other than prophylactic antiparasitic agents within 1 month of this study. The study protocol was approved by the Institutional Animal Care and Use Committee.

### Study Protocol

Jugular venipuncture was performed once on all animals using a 21-ga hypodermic needle attached to a 6-mL syringe. Immediately following sample acquisition, two 3.2% citrate blood tubes labeled “Tube A” and “Tube B” were filled by vacuum with 2.7 mL whole blood as recommended by the manufacturer to obtain a 1:9 citrate-to-blood ratio (4). Samples were gently inverted several times for mixing. The use of two citrate tubes in this case was for the purpose of running samples in duplicate to obtain within-sample variability data.

Samples were divided into two groups. NWB samples made up the control group, and CWB samples made up the test groups. Testing of CWB samples from each of the citrate tubes, labeled as Tube A and Tube B, was performed at three time points—immediately following 30-min rest period, 2 h and 4 h post-collection, termed as the baseline group, the 2-h group (Cit-2), and the 4-h group (Cit-4), respectively.

Viscoelastic coagulation testing was performed by a single user using the VCM Vet™ device per the manufacturer recommendations (8). Four VCM devices were used to run all of the study samples. Test cartridges (VCM Vet^TM^ Test Cartridge, Entegrion, Durham, NC) were prewarmed by use of the VCM heater plate (VCM Heater Plate, Entegrion, Durham, NC) to 37°C. For NWB samples, a 0.3 mL sample volume was introduced into a VCM test cartridge for analysis immediately after the citrate blood tubes were filled with a total time delay of <1 min between sample acquisition and sample introduction to the cartridge. All CWB samples were rested at room temperature for 30 min in an upright position in accordance with recommended sample handling practices for veterinary viscoelastic testing (4, 9). Directly prior to testing, sample recalcification was performed by adding 340 μL of CWB to a transfer vial containing 20 μL of 0.2 M calcium chloride *via* pipette (4). The sample was then immediately introduced into a VCM test cartridge for sample analysis. Samples were run for 60 min based on the maximum time allowed by the device software (8). All samples were maintained at 37°C during analysis *via* the VCM internal heating element, and sample drying was prevented by moisture strips contained within each cartridge.

Results were recorded on the device as a bitmap image containing the tracing and calculated viscoelastic parameters. Test results were reported using standard rotational thromboelastometry terminology including clotting time (CT), clot formation time (CFT), alpha angle (AA), maximum clot firmness (MCF), clot firmness amplitude at 10 (A10) and 20 (A20) min after clot formation, and clot lysis at 30 (LY30) and 45 (LY45) min.

### Statistical Analysis

All statistics were performed using commercially available software (JMP Pro v. 15.0.0, SAS Institute Inc., Cary, NC, USA). All parameters were evaluated using mixed analysis of variance (ANOVA) models with sample type (NWB, baseline, Cit-2, and Cit-4) as the fixed effect and individual patients as the random effect. When differences were detected, Tukey *post-hoc* comparisons were performed with least square means for the effect. Within-sample variability was investigated

for each parameter in the CWB group using coefficients of variation (CV%) using values pooled from all CWB data points. Assumptions of these models (linearity, normality of residuals, and homoscedasticity of residuals) and influential data points were assessed by examining standardized residual and quartile plots. All parameters except LY30 and LY45 were presented as means ± standard deviations. LY30 and LY45 were presented as median (range) and were evaluated using Friedman's test against sample types. A *post-hoc* Wilcoxon signed rank test was used. A *P* < 0.05 was considered significant for all comparisons. Bland-Altman plots were conducted with Prism 9 for Windows (GraphPad Software, San Diego, CA, USA).

## Results

**Figure 1:**
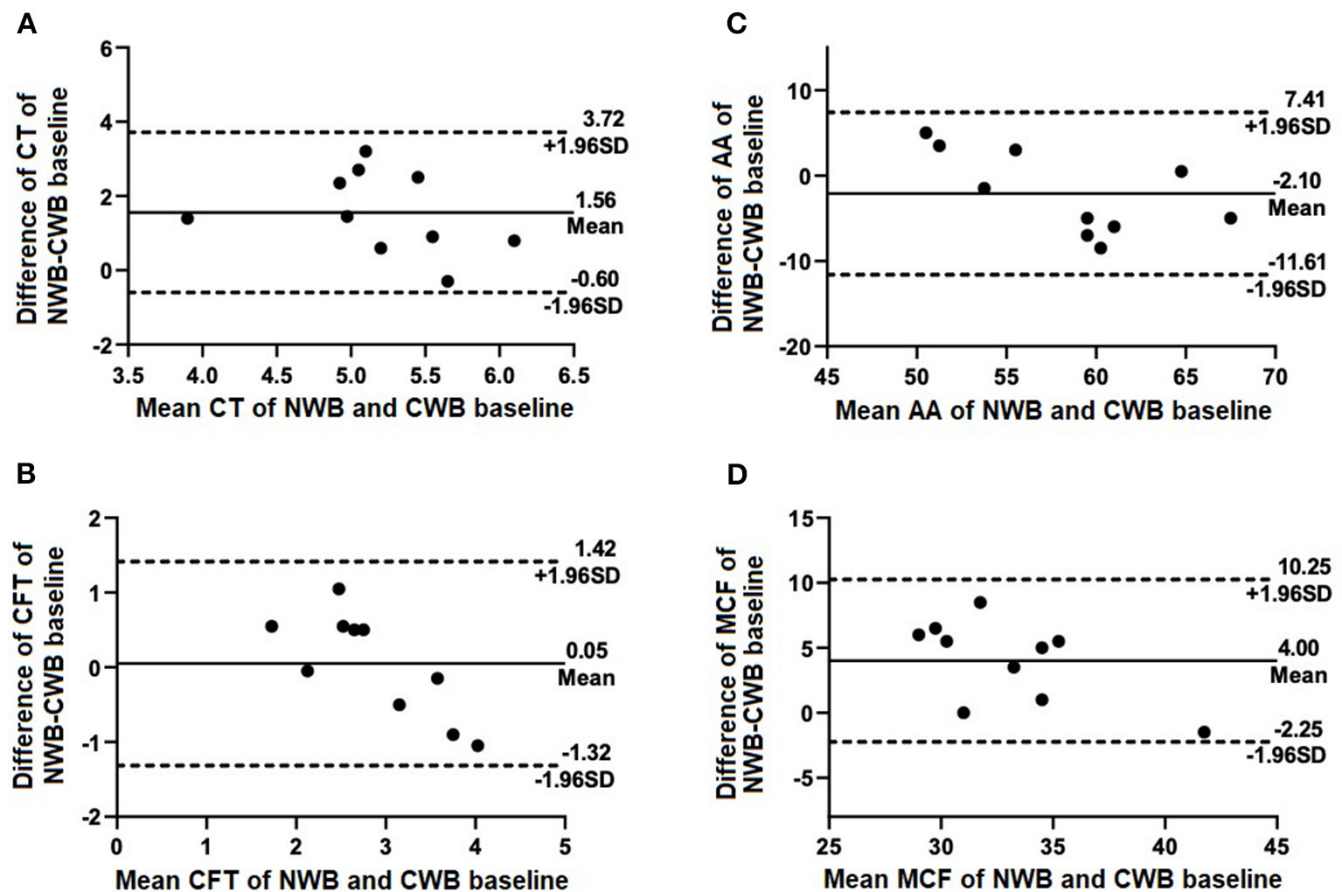
Selected Bland-Altman plots indicating biases of measured parameters of NWB (control) compared with CWB baseline. Mean difference (bias) is indicated by solid lines with dashed lines indicating 95% agreement interval of +/− standard deviations. Parameters included are **(A)** CT, **(B)** CFT, **(C)** AA, and **(D)** MCF.

**Figure 2:**
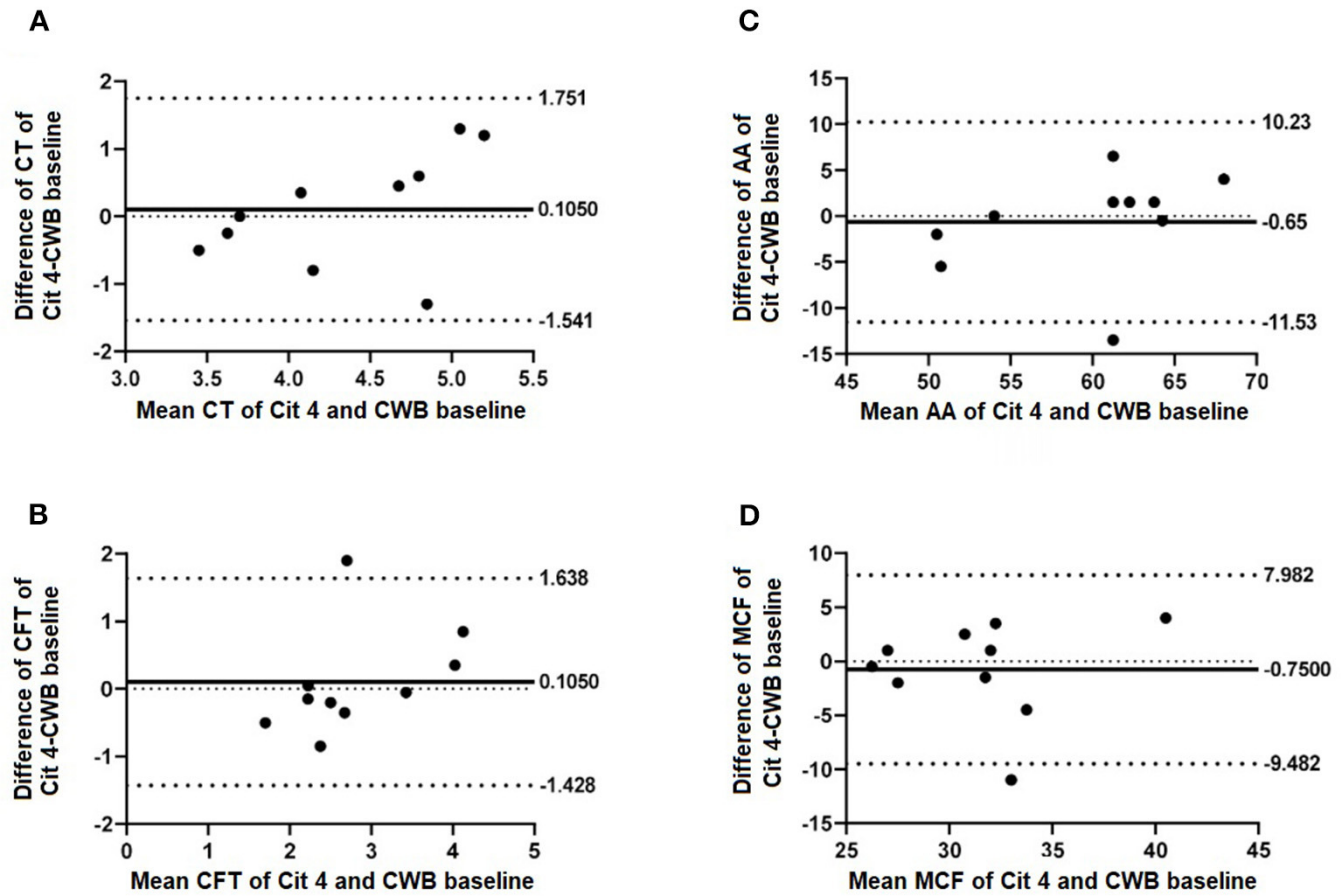
Selected Bland-Altman plots indicating biases of measured parameters of CWB baseline compared with 4 h rested CWB samples (Cit 4). Mean difference (bias) is indicated by solid lines with dashed lines indicating 95% agreement interval of +/− standard deviations. Parameters included are **(A)** CT, **(B)** CFT, **(C)** AA, and **(D)** MCF.

Results were obtained for a total of 10 NWB control runs and 20 CWB runs for each time point. No exclusions due to sample handling or testing errors were necessary. Results of NWB were compared to those of CWB baseline and are summarized in Table 1 and Figure 1. There were significant differences between NWB and baseline CWB in CT (*p* < 0.001) and MCF (*p* = 0.002). Comparison of CWB baseline to both other time points (Cit-2 and Cit-4) revealed no significant differences and are summarized in Table 2 and Figure 2. Within sample variability of CWB was determined for each parameter and is listed in Table 3.

**Table 1:** Results of NWB compared to CWB baseline.

	**NWB**	**CWB baseline**	***P*-value**
CT (min)	5.97 ± 0.66	4.41 ± 1.11	<0.0001*
CFT (min)	2.90 ± 0.51	2.85 ± 1.06	0.4139
α-angle (°)	57.30 ± 4.45	59.40 ± 7.76	0.1818
A10 (units)	22.60 ± 3.34	22.45 ± 5.31	0.3586
A20 (units)	29.70 ± 3.95	27.80 ± 5.52	0.1217
MCF (units)	35.10 ± 3.03	31.10 ± 5.42	0.0036*
LY30 (%)	99.50 ± 1.27	98.45 ± 4.84	0.6420
LY45 (%)	99.20 ± 1.62	97.35 ± 4.82	<0.0001*

*Results are listed as a mean with standard deviation. Asterisks identify values that are significantly different (defined as p <0.05)*.

**Table 2:** Results of CWB baseline compared to CWB rested for 2 h (Cit-2) and 4 h (Cit-4).

	**CWB baseline**	**Cit-2**	***P*-values of CWB baseline vs. Cit-2**	**Cit-4**	***P*-values of CWB baseline vs. Cit-4**
CT (min)	4.41 ± 1.11	4.68 ± 0.69	0.2010	4.31 ± 0.61	0.6102
CFT (min)	2.85 ± 1.06	3.00 ± 0.83	0.4139	2.72 ± 0.77	0.4139
α-angle (°)	59.40 ± 7.76	58.20 ± 6.10	0.1818	60.05 ± 5.97	0.1818
A10 (units)	22.45 ± 5.31	21.40 ± 4.27	0.3586	22.40 ± 4.06	0.3586
A20 (units)	27.80 ± 5.52	27.50 ± 4.70	0.1217	28.45 ± 4.37	0.1217
MCF (units)	31.10 ± 5.42	30.90 ± 4.52	0.9962	31.85 ± 4.64	0.8416
LY30 (%)	98.45 ± 4.84	98.55 ± 5.61	0.6420	99.70 ± 1.34	0.6420
LY45 (%)	97.35 ± 4.82	96.30 ± 7.89	0.4474	96.95 ± 5.58	0.4474

*Results are listed as a mean with standard deviation*.

**Table 3:** Within sample variation (CV, %) listed as a median.

	**Minimum**	**Maximum**	**Study CV (%)**	**VCM manufacturer CV (%)**
CT (min)	0	68.80	6.79	6.8
CFT (min)	0	32.93	7.90	N/A
α-angle (°)	0	19.46	2.36	4.7
A10 (units)	0	17.25	3.83	N/A
A20 (units)	0	23.14	4.49	N/A
MCF (units)	0	27.37	3.21	10.7
LY30 (%)	0	17.37	0.00	N/A
LY45 (%)	0	16.32	0.00	N/A

*Where known, the VCM manufacturer's within sample variation [CV (%)] is also listed. In instances for which the VCM manufacturer's CV is not known, “N/A” is listed instead*.

## Discussion

In the present study, we determined that citrated blood samples could be used in the cartridge based viscoelastic analyzer and that citrated sample results remained consistent up to 4 h after blood collection. This suggests that citrated samples could be taken upon presentation and could be run up to 4 h later, thereby providing a less constraining, clinically relevant alternative for its use. To the authors' knowledge, prior to this study only, NWB samples have been used with the point of care, cartridge-based viscoelastic device in accordance with the manufacturer's instructions.

Results obtained using NWB were consistent with, but not directly comparable to those using CWB. This was expected, in large part, due to previous studies that have shown a tendency toward hypercoagulability in citrated samples compared to NWB samples using TEG and TEM modalities (10, 11). Differences were found in the CT and MCF between NWB samples and baseline CWB samples. It is known that viscoelastic tests run on NWB and without activators are subject to more variabilities than their treated and activated counterparts. Such variabilities may include contact activation, operator-dependent factors, and other preanalytical factors (6, 10). These variabilities may explain the differences in CT and MCF between NWB and CWB samples. In the present study, CT values of NWB control samples were significantly longer than those of CWB samples. This echoes findings in both human and canine studies that have shown citrated samples' tendency toward hypercoagulability when compared with NWB samples used for TEG and rotational thromboelastometry (10, 12). Citration of blood largely inhibits thrombin formation and coagulation activation, but does not do so entirely (13, 14). Both of these mechanisms could explain the relative hypercoagulability of the present study's citrated CT results. On the other hand the MCF values of NWB showed a tendency toward hypercoagulability compared to the CWB samples. Further studies would be required to determine a potential cause of this finding. One potential explanation is a decrease in platelet numbers over time in citrated samples, which has been shown to occur in both EDTA and citrated blood samples (15). It has yet to be determined whether similar findings exist between NWB and CWB taken from patients with hemostatic disturbances, as the present study assessed only healthy patients.

No significant differences were found when comparing CWB baseline results to CWB results tested after both 2- and 4-h sample rest periods. These findings were unexpected, given previous work in both dogs and humans that demonstrated resting samples at room temperature for longer than 30 min prior to analysis caused a trend toward hypercoagulability (3, 4, 11–13, 16). The previously noted trend toward hypercoagulability could be explained by some level of *ex vivo* contact activation occurring over time with sample storage. Previously proposed mechanisms to explain this trend in stored samples include that citrated blood does not entirely inhibit thrombin formation or, later, activation of coagulation (13). One possible explanation for the lack of significant differences seen in viscoelastic parameters of citrated samples following increasing rest periods in this study is the lack of activators used for the point of care analyzer, despite the recommendation that activators be used for viscoelastic tests to reduce variability. Large efforts were made during this study to reduce sample variability factors. Further, the point of care viscoelastic analyzer of the present study utilizes different testing methods (two glass slides) than the traditional pin-and-cup methodology used in TEG and TEM, which could potentially account for the lack of hypercoagulability seen in samples with longer storage time. Further research is required to determine if this device is less prone to the variabilities with increasing sample storage time that are commonly seen using other modalities.

The current study demonstrated smaller within-sample variation than that of the analyzer's manufacturer (8). It is unknown whether the data collected by the VCM Vet manufacturer was compiled from testing completed by one operator or multiple. It is possible that the low within sample variation seen in the current study is because only one operator conducted the testing. The small sample size of dogs utilized in this study could also account for the lower within-sample variation. Another explanation for the present study's low within-sample variation is its use of only four analyzers, whereas the manufacturer used eight analyzers to generate their data. Previous studies involving humans and equids have shown that variability may exist between different operators conducting viscoelastic tests, thus reducing the number of operators may help to reduce within sample variation (5, 6, 17). Inter-operator variability has been studied using both TEG and rotational thromboelastometry, though inter-operator variability is currently not known for the VCM Vet device; however, a proposed advantage of the utilized cartridge-based viscoelastic testing system is that the cartridge system could reduce inter-operator variability. Another explanation for the current study's low within sample variation is strict standardization of the testing protocol that was made in accordance with the PROVETS guidelines, which were in part made to reduce variation in analyses caused by venipuncture site and technique as well as sample storage temperature.

There are several limitations to the current study. The utilized sample size of 10 dogs was sufficient for statistical analysis in this pilot study to determine significant differences between NWB and CWB samples, however, such a small sample size can lead to a type II statistical error. Secondly, this pilot study utilized blood samples from only healthy, intact female dogs. Though this allowed for uniformity of the sampled population, sex has been shown to affect results in some traditional methods of viscoelastic testing in people (2, 18). It is currently unknown whether sex affects viscoelastic testing in dogs. An additional limitation includes variabilities in venipuncture technique. Though all samples were collected with the same materials and from the jugular veins of test subjects, two different people were required to collect samples from the test subjects. Though the use of two different sample collectors could have influenced the overall statistical significance, this would not have altered sample consistency because each sample was collected at the same time and from the same venipuncture event. Variabilities from running of samples across multiple devices is an additional limitation, however, this effect was mitigated by using the same VCM Vet™ unit for each sample tube at each time point. Other efforts made to reduce variability included use of a single operator for all test analyses, the smallest number of analyzers possible, and the same equipment for all operator-dependent variables (i.e., venipuncture equipment, pipette, blood tube types).

In conclusion, recalcified CWB can be used with the cartridge based VCM Vet™ for reliable viscoelastic coagulation analysis up to 4 h after collection, however, new canine reference intervals will need to be established using this methodology. Further research will be necessary to assess consistency of recalcified CWB results in patients with coagulation derangement.

## Data Availability Statement

The original contributions presented in the study are included in the article/supplementary material, further inquiries can be directed to the corresponding author/s.

## Ethics Statement

The animal study was reviewed and approved by the Institutional Animal Care and Use Committee (IACUC) at the Louisiana State University School of Veterinary Medicine.

## Author Contributions

WY prepared and organized all materials used for blood collection, subsequent sample analysis, including recalcification of samples just prior to analysis, subsequently used the raw data to write and edit the manuscript, generate its tables, and also presented the original research at the 2020 International Veterinary Emergency and Critical Care Symposium. MRS ran all blood samples in the VCM Vet analyzer, contributed to the writing of the manuscript and was heavily involved in its editing, and also prepared all IACUC materials in order to obtain blood samples from the study dogs used. C-CL generated all of the statistics from the raw data and subsequently wrote all portions of the manuscript entailing statistical data and procedures. All authors contributed to the article and approved the submitted version.

## Conflict of Interest

The authors declare that the research was conducted in the absence of any commercial or financial relationships that could be construed as a potential conflict of interest.

## Publisher's Note

All claims expressed in this article are solely those of the authors and do not necessarily represent those of their affiliated organizations, or those of the publisher, the editors and the reviewers. Any product that may be evaluated in this article, or claim that may be made by its manufacturer, is not guaranteed or endorsed by the publisher.

## Abbreviations

A10: amplitude at 10 min after clot formation
A20: amplitude at 20 min after clot formation
AA: alpha angle
CFT: clot formation time
CT: clotting time
CV%: coefficient of variation
CWB: citrated whole blood
LY30: lysis at 30 min
LY45: lysis at 45 min
MCF: maximum clot firmness
NWB: native whole blood
TEM: thromboelastometry
TEG: thromboelastography.

## References

[1] Cohen T Haas T Cushing MM. The strengths and weaknesses of viscoelastic testing compared to traditional coagulation testing. Transfusion. (2020) 60:S21–8. 10.1111/trf.16073 33089934

[2] McMichael MA Smith SA. Viscoelastic coagulation testing: technology, applications, and limitations. Vet Clin Pathol. (2011) 40:140–53. 10.1111/j.1939-165X.2011.00302.x 21446994

[3] Ralph AG Brainard BM Pittman JR Babski DM Koenig A. Effects of rest temperature, contact activation, and sample technique on canine thromboelastography. J Vet Emerg Crit Care. (2012) 22:320–6. 10.1111/j.1476-4431.2012.00730.x 22702438

[4] Goggs R Brainard B de Laforcade AM Flatland B Hanel R McMichael M. Partnership on Rotational ViscoElastic Test Standardization (PROVETS): evidence-based guidelines on rotational viscoelastic assays in veterinary medicine. J Vet Emerg Crit Car. (2012) 22:320–6. 10.1111/vec.12144 24422679

[5] Anderson L Quasim I Steven M Moise SF Shelley B Schraag S. Interoperator and intraoperator variability of whole blood coagulation assays: a comparison of thromboelastography and rotational throsmboelastometry. J Cardiothorac Vasc Anesth. (2014) 28:1550–7. 10.1053/j.jvca.2014.05.023 25267692

[6] Buriko Y Drobatz K Silverstein DC. Establishment of normal reference intervals in dogs using a viscoelastic point-of-care coagulation monitor and its comparison with thromboelastography. Vet Clin Pathol. (2020) 49:657–573. 10.1111/vcp.12926 33368568

[7] Rosati T Jandrey KE Burges JW Kent MS. Establishment of a reference interval for a novel viscoelastic coagulometer and comparison with thromboelastography in healthy cats. Vet Clin Pathol. (2020) 49:660–4. 10.1111/vcp.12916 33207006

[8] VCM Vet ™ Operator's Manual. (2019). Durham, NC: Entegrion Inc.

[9] Bowbrick VA Mikhailidis DP Stansby G. The use of citrated whole blood in thromboelastography. Anesth Analg. (2000) 90:1086–8. 10.1097/00000539-200005000-00015 10781457

[10] Silverberg E Tornqvist F Kander T Bengzon J Soloman C Bonnevier J. Comparison of citrated and fresh whole blood for viscoelastic testing during elective neurosurgery. Thromb Res. (2017) 156:73–9. 10.1016/j.thromres.2017.05.033 28601642

[11] Smith SA McMichael M Galligan A Gilor S Hoh CM. Clot formation in canine whole blood as measured by rotational thromboelastometry is influenced by sample handling and coagulation activator. Blood Coagul Fibrinolysis. (2010) 21:692–702. 10.1097/MBC.0b013e32833e9c47 20739876

[12] Zambruni A Thalheimer U Leandro G Perry D Burroughs A. Thromboelastography with citrated blood: comparability with native blood, stability of citrate storage and effect of repeated sampling. Blood Coagul Fibrinol. (2004) 15:103–7. 10.1097/00001721-200401000-00017 15166952

[13] Camenzind V Bombeli T Seifert B Jamnicki M Popovic D Pasch T. Citrate storage affects Thromboelastograph analysis. Anesthesiology. (2000) 92:1242–9. 10.1097/00000542-200005000-00011 10781268

[14] Papp AC Hatzakis H Bracey A Wu KK. Development of a blood collection and processing system suitable for multicenter hemostatic studies. Thromb Haemost. (1989) 61:15–9. 10.1055/s-0038-1646519 2526384

[15] Weber D Nakashima M. 198 Platelet count stability in sodium citrate-anticoagulated whole blood samples. Am J Clin Pathol. (2018) 149:S84–5. 10.1093/ajcp/aqx121.197

[16] Wiinberg B Jensen AL Rojkjaer R Johansson P Kjelgaard-Hansen M Kristensen AT. Validation of human recombinant tissue factor-activated thromboelastography on citrated whole blood from clinically healthy dogs. Vet Clin Pathol. (2005) 34:389–93. 10.1111/j.1939-165X.2005.tb00066.x 16270265

[17] Thane K Bedenice D Pacheco A. Operator-based variability of equine thromboelastography. J Vet Emerg Crit Care. (2017) 27:419–24. 10.1111/vec.12610 28520166

[18] Horlocker TT Schroeder DR. Effect of age, gender, and platelet count on Sonoclot coagulation analysis in patients undergoing orthopedic operations. Mayo Clin Proc. (1997) 72:214–9. 10.4065/72.3.214 9070195

